# Auditory Charles Bonnet Syndrome in a Geriatric Patient with Hearing Loss and Depressive History: A Case Report and Multidisciplinary Approach

**DOI:** 10.1192/j.eurpsy.2025.1754

**Published:** 2025-08-26

**Authors:** A. Romero Teruel, A. Vives-Luengo, M. Aledo-Serrano, B. Franco-Lovaco

**Affiliations:** 1 Department of Psychiatry, Hospital Dr. Rodríguez Lafora; 2 Department of Neurology, Hospital Universitario La Paz, Madrid, Spain

## Abstract

**Introduction:**

We present the case of an 88-year-old woman with severe hearing loss and a history of hypertension, non-valvular atrial fibrillation (AF), hypothyroidism, and depressive episodes, admitted following a medication overdose in a context of depressive ideation. The patient reported auditory hallucinations, hearing the voice of her deceased mother; however, she did not exhibit delusional interpretations regarding these experiences, suggesting auditory Charles Bonnet syndrome. This rare phenomenon is primarily described in older patients without psychotic disorder and requires an interdisciplinary approach for appropriate management.

**Objectives:**

To describe the clinical progression of a probable case of auditory Charles Bonnet syndrome and analyze the importance of a multidisciplinary approach, particularly in coordination with neurology, to achieve optimal diagnosis and treatment in a geriatric context.

**Methods:**

The case was addressed through a detailed psychiatric evaluation focused on psychopathological assessment and structured interviews to evaluate affective, cognitive, and behavioral symptoms. Neuropsychological assessment included the Phototest and Clock Drawing Test to rule out advanced cognitive impairment, as well as a cranial CT scan, which showed no significant abnormalities. The neurology consultation evaluated cognitive status, hearing loss, and its impact on the patient’s psychological state, while also ruling out other neurological disorders.

**Results:**

The patient showed favorable progress during admission, with mood stabilization and reduced anxiety. She exhibited long-standing depressive symptoms, including feelings of guilt and burden on her family. She acknowledged hearing her mother’s voice calling her and was able to critically assess the unreal nature of these hallucinations. The neurology consultation confirmed significant hearing loss, which contributed to her isolation and, possibly, to the development of auditory hallucinations (due to the lack of sensory input). No signs of advanced cognitive impairment were found.

**Conclusions:**

This case highlights the importance of a multidisciplinary approach in managing elderly patients with unusual hallucinatory phenomena. Once psychotic symptoms were ruled out by psychiatry, the neurology consultation was essential to exclude cognitive impairment and contextualize the auditory hallucinations in relation to the patient’s hearing loss and emotional burden. The integrated intervention with her family facilitated a more comprehensive approach, including family support and psychoeducation, adjustments in psychopharmacological treatment, and recommendations to improve quality of life (including emphasizing the use of hearing aids) and social support; demonstrating the effectiveness of collaboration between psychiatry and neurology.

**Disclosure of Interest:**

None Declared

